# Research and Remedies

**Published:** 1912-06-29

**Authors:** 


### RESEARCH AND REMEDIES.

### Work and Progress on Leprosy.

A short time ago a considerable stir was made in the lay press over the fact that leprosy is not a notifiable disease in this country, and that patients suffering from it are allowed to mingle with their fellow-citizens without let or hindrance. That there are imported cases of leprosy in England no one doubts; though it has yet to be established that the infection has been transmitted from them to any uninfected person. The possibility of such an event in the future cannot, of course, be disproved; and if some recent researches are confirmed, the obscure problem of the transmission of this disease may be settled in such a way as to show that the possibility is quite a probability. The theory of some recent investigators is that the bacillus of leprosy may be transmitted from the leprous to the healthy by means of parasitic animals such as the bed-bug. The latest adherent to this view is Dr. Long, principal medical officer of Basutoland, whose researches confirm those of others in several respects. Inasmuch as the bug is a creature not confined to those regions where leprosy is endemic, the immunity which most temperate climates enjoy from leprosy cannot be regarded as absolute.

As regards treatment, a careful summary of work at Robben Island is given in the *Journal of Tropical Medicine and Hygiene* by Dr. Sanders, who has given an extensive trial to many of the plans advocated from time to time. Thus a diet of fruit and vegetables exclusively was given to ten patients. At first it did them good by relieving them of digestive disturbances due to eating a too liberal allowance of meat; but the leprosy was not benefited, and most of the patients went steadily downhill. Then the (locally) praised "Jagger's treatment" was tried, and shown to be equally without value. Thyroid gland, vaccines, iodides, organic arsenic compounds, and several other drugs were also complete failures. Radium proved to be capable of inhibiting the growth of lepra bacilli *in vitro*; but unfortunately *in corpore vili* it destroyed the living cells at a greater rate than the bacilli, so that the remedy was worse than the disease. Chaulmoogra oil, however, gave better results. This remedy is no new one, but Dr. Sanders is convinced that it is the only one which does good. He holds that when properly used it produces marked improvement in 30 to 40 per cent., and indeed an absolute cure in a certain proportion of cases. The treatment must be persevered with regularly for five years or so; and every other resource must be utilised as well, including surgical intervention in appropriate cases.

### Diagnosis of Suppuration in the Maxillary Antrum.

The labours of numerous rhinologists have brought home to the medical profession the frequency of suppuration in the various accessory sinuses of the nose, and the obscurity of the symptoms in many cases. In particular, the influenza bacillus seems to have a partiality for settling in these cavities, and for defying dislodgement. Not only are the evidences of such sinusitis often obscure; but even when there is good ground for suspecting suppuration of this kind, it is often difficult to be sure exactly which regions are involved. Thus acute suppuration in the maxillary antrum often mimics with some exactness the symptoms of frontal or ethmoid sinusitis. In transillumination a very valuable aid to diagnosis exists; but accumulating experience shows that it does not do to trust too absolutely even to this method. Sometimes a maxilla which seems definitely opaque is found to contain no pus; and sometimes, too, one which is translucent turns out to be full of pus after all: so that neither negatively nor positively is the test entirely diagnostic.

Impressed with these limitations of the transillumination method, Dr. Watson-Williams advocates in the *Journal of Laryngology* a return to the old plan of aspiration of the antrum through the middle meatus in all doubtful cases. This he performs, not with a trocar and cannula, but with a special suction syringe provided with a narrow bore needle. After cocainisation, the procedure causes almost no pain. The syringe is nearly filled with distilled water, which is quickly injected through the exploring needle into the antrum, and then at once sucked back into the syringe. Thus the nature of the contents of the antrum is discovered, and the diagnosis of suppuration can be made when all other methods have failed to clinch it.

### Radium for Laryngeal Papilloma.

With the empirical use of radium for all kinds of growths and tumours lately become fashionable, there is a grave risk that results will be attributed to this agent for which, it may be, other causes are really responsible. But two remarkable cases, described in detail in the New York *Medical Record*, seem so clearly to show that radium can influence that very obstinate and troublesome condition, papilloma of the vocal cords, that further trial of the remedy can be confidently advised. A patient who suffered from perpetually recurring attacks of these innocent growths was under the care of three successive American surgeons, and required an operation every six months for forty-seven years. At last, four years ago, it was decided to try radium; the growths were extensive, but not malignant. After treatment no further operation was required for three years, and the comfort of the patient was greatly enhanced. Fortified by this encouraging, though not by any means perfect result, the radium treatment was then applied to a young woman who had already had two operations in nine months and was suffering from a rapid and very extensive recurrence. In this case it was found necessary to do a temporary tracheotomy in order to get the radium capsule into position; but after one exposure and no further treatment the cords were restored to a perfectly normal state, and six months later the patient sang at a meeting of a medical society. Two more cases are now under treatment and are said to be progressing very satisfactorily.

### Salvarsan for Bilharziasis.

Not long ago a physician practising in Egypt, whose name suggests a Levantine origin, published a paper claiming that salvarsan cures bilharziasis, the parasitic disease which accounts for so much pain, invalidism, and even death in Egypt. This pronouncement was accepted by Ehrlich as one more proof of the value of his remedy. The premises from which the conclusion was drawn are severely criticised in the *Lancet* by Dr. Day and Mr. Richards, of the Kasr-el-Ainy Hospital, Cairo, who lay down three conditions which the ideal treatment of bilharziasis must fulfil. It must destroy the worms; it must cause or allow complete expulsion of the eggs; and it must destroy the latter so that they cease to be a danger to others. Salvarsan, they say, was not shown in the paper criticised to do any of these things, but actually to fail in respect of the second condition. The authors decided, in view of the enormous importance of bilharzia in Egypt, to carry out independent tests to settle the matter if possible. Three cases were treated, not one of which benefited in the slightest degree from the treatment. The cases were watched and followed up with great care, and blood examinations were made to help in determining whether the condition of the patients had altered. It is concluded that salvarsan is absolutely valueless as a remedy for bilharziasis, and that it should not be used or recommended for this purpose.

### Hernia Operations.

At the present time surgical opinion has veered round almost to the opposite of what it was ten years ago in respect of one point in the ætiology of inguinal hernia. Thanks to the writings of Mr. R. W. Murray and others, it is now believed that in most cases of inguinal hernia the sac is preformed, not acquired; that is, it exists from birth owing to failure in complete closure of the funicular process, and is not formed by protrusion through the inguinal canal of a process of abdominal peritoneum. Adopting this view, Mr. E. W. Roughton pleads for bilateral operation whenever a radical cure is being done for a child or a young adult. He argues that thus a sac may be found on the supposed sound side which might otherwise become the seat of a future hernia. If none is found the wound can be sewn up, and no harm is done. This course seems to omit to take into account the natural objection of patients to unnecessary incisions, though Mr. Roughton's figures—ten cases of latent sac found as against only eight in which there was none—certainly support his contention, as far as such a small series of cases can.

